# MSA‐MaxNet: Multi‐Scale Attention Enhanced Multi‐Axis Vision Transformer Network for Medical Image Segmentation

**DOI:** 10.1111/jcmm.70315

**Published:** 2024-12-20

**Authors:** Wei Wu, Junfeng Huang, Mingxuan Zhang, Yichen Li, Qijia Yu, Qi Zhao

**Affiliations:** ^1^ School of Computer Science and Software Engineering University of Science and Technology Liaoning Anshan China; ^2^ School of AI and Advanced Computing, XJTLU Entrepreneur College (Taicang) Xi'an Jiaotong‐Liverpool University Suzhou China; ^3^ School of Electronic and Information Engineering University of Science and Technology Liaoning Anshan China; ^4^ Wenzhou Institute University of Chinese Academy of Sciences Wenzhou China

**Keywords:** CNN, medical image segmentation, multi‐scale, pathological changes, self‐attention

## Abstract

Convolutional neural networks (CNNs) are well established in handling local features in visual tasks; yet, they falter in managing complex spatial relationships and long‐range dependencies that are crucial for medical image segmentation, particularly in identifying pathological changes. While vision transformer (ViT) excels in addressing long‐range dependencies, their ability to leverage local features remains inadequate. Recent ViT variants have merged CNNs to improve feature representation and segmentation outcomes, yet challenges with limited receptive fields and precise feature representation persist. In this work, we propose MSA‐MaxNet. Specifically, our model utilises an encoder–decoder structure, using MaxViT blocks that apply multi‐axis self‐attention (Max‐SA) as the encoder for local and global feature extraction. To restore the feature map's spatial resolution during upsampling operations, a symmetric MaxViT block–based decoder and upsampling layers are employed. To address the feature mismatches in the skip connections of UNet architecture, we introduce convolutional block attention module (CBAM). Furthermore, we design a multi‐scale convolutional block attention module (MCBAM) based on CBAM, which utilises multi‐scale features to enhance feature representation and refine the skip connection. We evaluate the segmentation performance of MSA‐MaxNet on three publicly available medical imaging datasets, including Synapse for multi‐organ segmentation, ACDC for cardiac analysis and Kvasir‐SEG for gastrointestinal polyp detection. Notably, MSA‐MaxNet achieves state‐of‐the‐art (SOTA) Dice scores of 85.59% and 95.26% on Synapse and Kvasir‐SEG datasets, respectively, with 40.28 M parameters. Additionally, we introduce two smaller versions of MSA‐MaxNet to meet the demands of various scenarios. In summary, our work provides a robust framework for diverse medical imaging tasks, offering potential applications in early cancer detection, cardiovascular disease diagnosis and comprehensive organ‐level assessments.

**Abbreviations**
ACDC: automated cardiac diagnosis challenge
CBAM: convolutional block attention module
CNNs: convolutional neural networks
CT: computed Tomography
DSC: Dice similarity coefficient
FFN: feedforward network
HD95: 95 th percentile of the Hausdorff distance
LV: left ventricle
Max‐SA: multi‐axis self‐attention
MCBAM: multi‐scale convolutional block attention module
MLP: multi‐layer perceptron
MRI: magnetic resonance imaging
Myo: myocardium
RV: right ventricle
Synapse: Synapse multi‐organ segmentation dataset
ViT: vision transformer

## 1 Introduction

Medical image segmentation plays a critical role in clinical diagnosis and treatment planning, enabling precise delineation of anatomical structures. Advanced medical imaging techniques, such as CT and MRI, are instrumental in the diagnosis and management of a broad range of serious medical conditions [1, 2]. Traditionally, medical image segmentation methods have relied on filter‐based techniques, such as edge detection [3] and mathematical morphology [4]. However, these methods often struggle with limited feature extraction capabilities and unstable segmentation accuracy, making them challenging to deploy effectively in practical scenarios [5].

In recent years, owing to the continuous advancement in computational capabilities, machine learning techniques, particularly deep learning techniques, have found application in various bioinformatics domains, including the analysis of computational toxicology [6, 7, 8], miRNA–lncRNA interactions prediction [9, 10, 11], metabolite–disease associations prediction [12, 13], and remote health monitoring [14, 15, 16]. These studies not only promote the development of biology and medicine but also provide vital references for the implementation of deep learning in additional fields, such as the modelling of biological system dynamics and decision‐making processes [17, 18, 19]. Currently, deep learning has been extensively applied to medical image segmentation, with numerous papers documenting its success in this field [20, 21, 22]. Notably, the architecture of U‐Net [23] and its variants [24, 25, 26] have become the backbone of many medical image segmentation models due to their effective encoder–decoder structure with skip connections that capture both spatial and contextual information [27]. However, convolutional neural network (CNN)–based architectures inherently have limitations in capturing wide‐ranging context due to the localised receptive fields of convolutional layers [28].

To address this limitation, combining ViT with U‐Net architectures has emerged as a promising approach to leverage the strengths of both models [29]. Models like TransUNet [30] integrate ViT blocks into U‐Net architecture to capture wide‐ranging dependencies. However, its reliance on a large stack of ViT modules significantly increases the number of parameters (∼100 M), thereby raising computational complexity, training costs and the risk of overfitting. Swin‐UNet [31] and GCCSwin‐UNet [32] attempt to address this issue by introducing different ViT variants. However, limited by the scope of microscopic attention, they struggle to capture long‐range dependencies across large‐scale targets. Recently, AgileFormer [33] introduced deformable attention mechanisms to adaptively capture features of varying shapes and sizes. However, the added complexity of learning additional sampling point positions not only increases model uncertainty but also significantly raises the parameter count (∼120 M). This underscores a key limitation in current models, which find it challenging to strike an optimal balance between parameter efficiency and the ability to capture features across varying spatial scales. Following the predicament, we turn our focus to Max‐SA mechanism [34], which utilises block‐level attention to extract information from neighbouring regions and employs a sparse, grid‐based self‐attention strategy to efficiently capture dependencies among distant features. This approach demonstrates greater advantages in handling targets with large‐scale variations and irregular boundaries. Furthermore, this design applies self‐attention only at specific positions, enabling efficient information extraction while maintaining linear complexity, foreboding a promising prospect. Therefore, we aim to fully leverage the advantages of Max‐SA mechanism within the UNet architecture, enabling more effective multi‐scale feature extraction and enhancing inter‐layer feature interaction.

In the context of ViT‐UNets, we argue that skip connections act as a double‐edged sword, a factor that may have been overlooked in previous years' research. While skip connections help preserve spatial information and mitigate vanishing gradient problems, they can also introduce redundant information, such as noise from low‐level feature maps, and feature mismatches between the encoder and decoder may occur, resulting in suboptimal fusion and potential loss of important information. Models, such as UCTransNet [35], EMCAD [36] and HieraUNet [37], attempted to improve feature fusion in the decoder by introducing multi‐level or attention‐based mechanisms within skip connections. However, these designs often face detail loss and slower inference issues. Therefore, optimising skip connections has become a crucial focus in our work. The channel and spatial attention mechanisms [38] are capable of adaptively focusing on critical information within feature channels and spatial dimensions, effectively reducing redundancy and interference. However, inconsistencies between features at different stages during skip connections remain a challenge, necessitating more advanced attention mechanisms for optimisation. To address this, we propose MCBAM, which computes weights and extracts attention from adjacent stages to suppress noise while precisely calibrating features at the current level, thereby improving the accuracy of feature representation. Furthermore, as features from different stages vary in dimensionality, encompassing either more abstract semantic information or finer details, these multi‐scale features complement each other across levels, further correcting deficiencies in feature extraction at the current stage.

Based on the factors discussed above, we propose MSA‐MaxNet, a model that integrates multi‐axis vision transformer (MaxViT) blocks to capture holistic features through Max‐SA mechanism, while employing MCBAM to refine skip connections, leading to more precise and efficient segmentation results. The main contributions of this work are summarised as follows:

1. We incorporate MaxViT blocks into both the encoder and decoder stages. By embedding these blocks, which leverage Max‐SA mechanisms, we significantly enhance the model's ability to capture both local details and global contexts, addressing long‐range dependencies and complex spatial hierarchies in the input CT and MRI images.
2. We propose MCBAM, which aggregates and adjusts multi‐scale features from different encoder stages using channel and spatial attention mechanisms, enhancing feature representation and fusion by maintaining spatial information and providing refined contextual awareness.
3. We validate our approach on three distinct medical segmentation datasets (Synapse, ACDC and Kvasir‐SEG), demonstrating that MSA‐MaxNet achieves new SOTA performance. This outstanding performance across multiple datasets underscores the model's robustness, efficiency and its potential for advancing precise segmentation in a wide range of biomedical and pathological applications.

## 2 Materials and Methods

### 2.1 Overall Architecture

**FIGURE 1:**
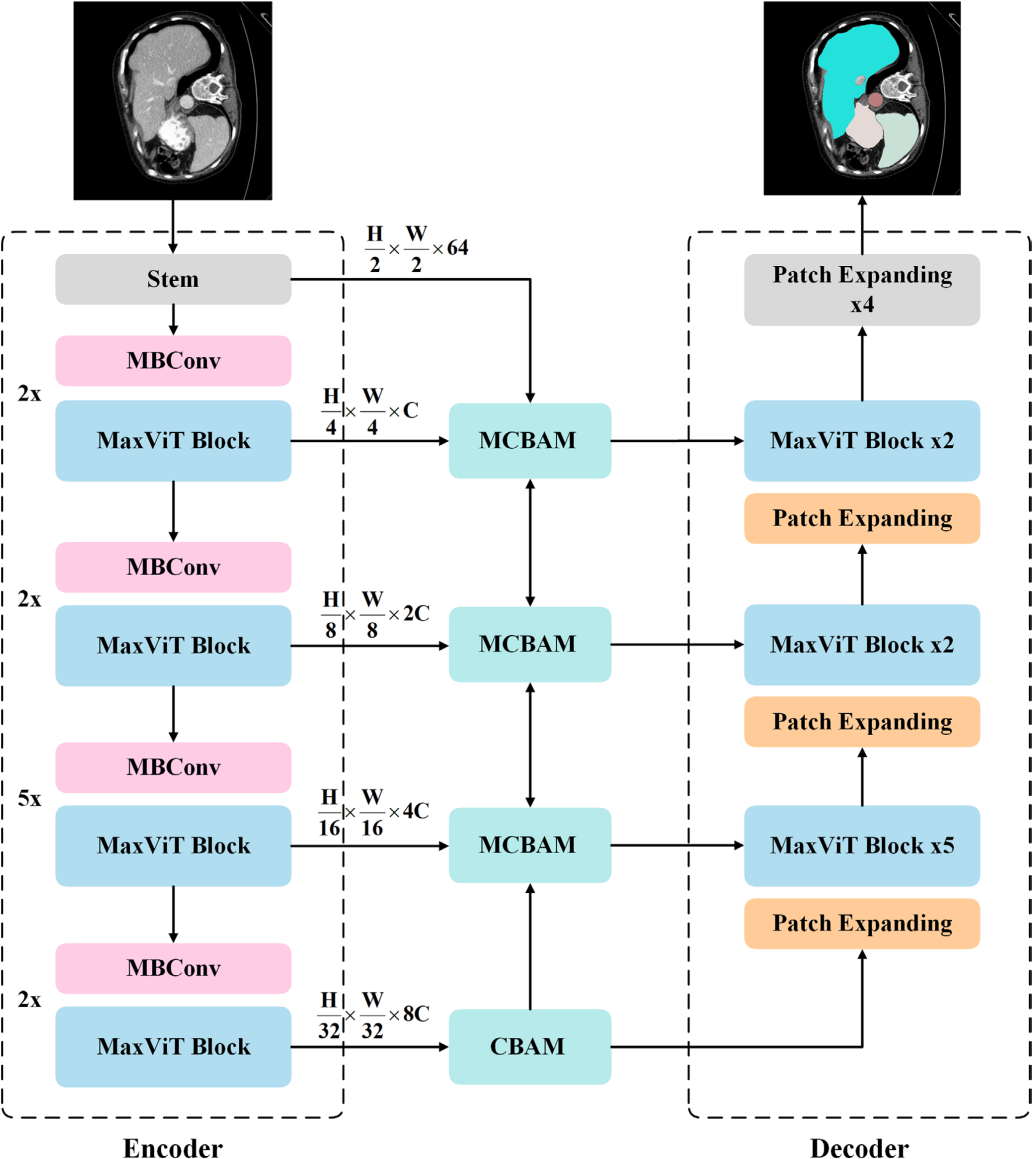
The architecture of MSA‐MaxNet.

The overall architecture of the proposed MSA‐MaxNet is illustrated in Figure 1. MSA‐MaxNet consists of an encoder, decoder, MCBAM, CBAM and skip connections. This network begins with the input image passing through a stem layer, which splits the input image into nonoverlapping patches, resulting in features with dimensions $\frac{H}{2}\times \frac{W}{2}\times 64$. The encoder comprises a series of MBConv layers followed by MaxViT blocks, which are repeated multiple times at each stage to progressively capture and encode features from the input image. Specifically, MBConv layers utilise depthwise separable convolutions, serving both to perform 2× downsampling and to enhance feature extraction without altering the spatial resolution. MCBAM is a module that receives input features from the corresponding encoder stage, as well as features from one stage above and one stage below within the encoder hierarchy, processing these multi‐scale features to generate a refined feature map that encapsulates the comprehensive contextual information. After the final MaxViT block in the encoder, we utilise a CBAM to refine the feature representation before the decoder starts to reconstruct the segmented image. The decoder consists of patch expanding layers interspersed with MaxViT blocks to gradually restore the resolution of features, which reshape and perform 2× upsampling of resolution. Skip connections are used to fuse the upsampled features with corresponding outputs from MCBAM at each stage of upsampling. Finally, the last patch expanding layer is used to perform 4× upsampling to restore features to the original resolution (*W × H*), producing an image with pixel‐level segmentation predictions. We elaborate on each component in the following subsections.

### 2.2 MaxViT Block

**FIGURE 2:**
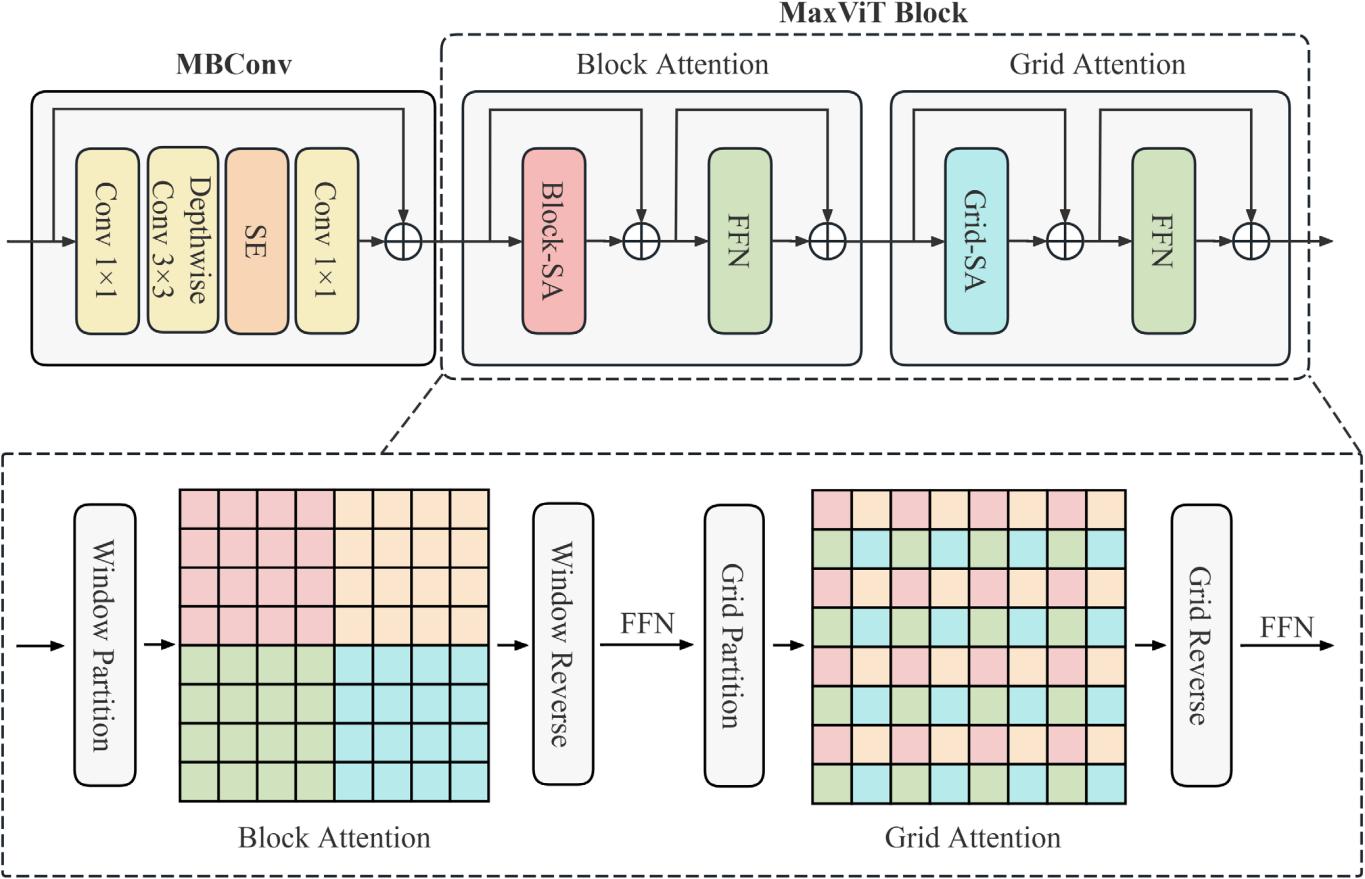
The diagram illustrates the detailed structures of MBConv and MaxViT block.

As shown in Figure 2, MaxViT block comprises two main sequential components: block attention and grid attention. Both components partition the feature map into segments. Block attention divides it into nonoverlapping windows, while grid attention divides it into a grid structure. Self‐attention mechanisms are applied within these segments to capture local dependencies in block attention and long‐range dependencies in grid attention. This process refines local features and integrates global context, enhancing feature representations. After self‐attention, the segments are merged back to their original shape. Notably, block attention and grid attention have a similar structure, and their detailed workflows are illustrated in Figure 2. Each consists of a self‐attention mechanism, Block‐SA and Grid‐SA, respectively, followed by a feedforward network (FFN). Mathematically, the procedures in Block‐SA and Grid‐SA can be expressed as follows:

(1) $$\text{Block}:\left(H,W,C\right)\to \left(\frac{H}{P}\times P,\frac{W}{P}\times P,C\right)\to \left(\frac{\mathrm{HW}}{P^{2}},P^{2},C\right),$$

(2) $$\text{Grid}:\left(H,W,C\right)\to \left(G\times \frac{H}{G},G\times \frac{W}{G},C\right)\to \underset{\text{swapaxes}\;\left(\text{axis}\;1=-2,\text{axis}\;2=-3\right)}{\underbrace{\left(G^{2},\frac{\mathrm{HW}}{G^{2}},C\right)\to \left(\frac{\mathrm{HW}}{G^{2}},G^{2},C\right)}},$$

where we define operation Block with parameter *P* as partitioning the input image/feature $x\in R^{H\times W\times C}$ into nonoverlapping blocks, each of size *P* × *P*. Similarly, we define Grid operation with parameter *G* as dividing the input feature into *G* uniform grids, where each grid is of adaptive size $\frac{H}{G}\times \frac{W}{G}$. In Block operation, after partitioning into windows, the block dimensions are gathered onto the spatial dimension (i.e., −2 axis). Unlike Block operator, Grid operation requires an additional transpose to place the grid dimension onto the assumed spatial axis (i.e., −2 axis). Additionally, the calculations for all block and grid attention utilise relative attention, which can be formulated as:

(3) $$\text{RelAttention}\left(Q,K,V\right)=\text{softmax}\left({\mathrm{QK}}^{T}/\sqrt{d}+B\right)V,$$

where $Q,K,V\in R^{\left(H\times W\right)\times C}$ are the query, key and value matrices, and *d* is the hidden dimension. *B* represents the relative positional bias, which is parameterised by a matrix $\hat{B}\in R^{\left(2H-1\right)\left(2W-1\right)}$. To reverse the block and grid partition after the calculation of relative attention, we define Unblock and Ungrid operations, respectively. The procedure of the multi‐axis attention module can be clearly explained as follows. Given an input tensor $x\in R^{H\times W\times C}$, the local Block Attention can be expressed as:

(4) $$\begin{array}{c} x\leftarrow x+\text{Unblock}\left(\text{RelAttention}\left(\text{Block}\left(\mathrm{LN}\left(x\right)\right)\right)\right)\\ x\leftarrow x+\mathrm{MLP}\left(\mathrm{LN}\left(x\right)\right) \end{array}.$$

While the global, dilated Grid Attention module is formulated as:

(5) $$\begin{array}{c} x\leftarrow x+\text{Ungrid}\left(\text{RelAttention}\left(\text{Grid}\left(\mathrm{LN}\left(x\right)\right)\right)\right)\\ x\leftarrow x+\mathrm{MLP}\left(\mathrm{LN}\left(x\right)\right) \end{array},$$

where LN denotes layer normalisation, and MLP is a standard multi‐layer perceptron network consisting of two linear layers: $x\leftarrow W_{2}\text{GELU}\left(W_{1}x\right)$.

### 2.3 Encoder

After passing through the stem layer, the *C*‐dimensional tokenised inputs with the resolution of $\frac{H}{2}\times \frac{W}{2}$ are fed into a series of MBConv layers followed by a MaxViT block. The encoder is structured into four stages, each comprising a sequence of MBConv layers and MaxViT blocks. In the first, second and fourth stages, the combination of one MBConv layer followed by one MaxViT block is repeated twice, whereas in the third stage, this combination is repeated five times. Throughout the encoder, the spatial resolution of the feature maps is progressively reduced from $\frac{H}{4}\times \frac{W}{4}\times C$ to$\frac{H}{32}\times \frac{W}{32}\times 8C$. Specifically, in each stage, the first MBConv performs depthwise separable convolutions to downsample the spatial resolution by a factor of 2, reducing the spatial dimensions while doubling the number of channels. The subsequent MBConv in each stage, while not involved in downsampling, continues to enhance feature extraction and refine the feature representations. Following each MBConv, a MaxViT block is applied. The MaxViT block receives the output of MBConv and applies Max‐SA mechanisms to capture both local and global features, further enhancing the feature representation as it progresses through the stages.

MBConv layer begins with a 1 × 1 pointwise convolution (Conv) to increase feature dimensionality, followed by a 3 × 3 depthwise convolution (DWConv) to capture spatial information. Notably, Norm denotes BatchNorm, both Conv and DWConv are followed by BatchNorm and GELU activation. A squeeze–excitation (SE) layer recalibrates channel‐wise features, and another 1 × 1 pointwise convolution (Proj) is applied to down‐project the number of channels. This process downsamples the spatial resolution by 2× while doubling the feature dimension. Assuming x to be the input feature, the MBConv layer without downsampling is represented as:

(6) $$x\leftarrow x+\text{Proj}\left(\mathrm{SE}\left(\text{DWConv}\left(\text{Conv}\left(\text{Norm}\left(x\right)\right)\right)\right)\right).$$

For the first MBConv block in each stage, downsampling is achieved using a stride‐2 3 × 3 depthwise convolution, with the shortcut branch involving pooling and channel projection:

(7) $$x\leftarrow \text{Proj}\left(\text{Pool}2D\left(x\right)\right)+\text{Proj}\left(\mathrm{SE}\left(\text{DWConv}↓\left(\text{Conv}\left(\text{Norm}\left(x\right)\right)\right)\right)\right).$$

### 2.4 CBAM

**FIGURE 3:**
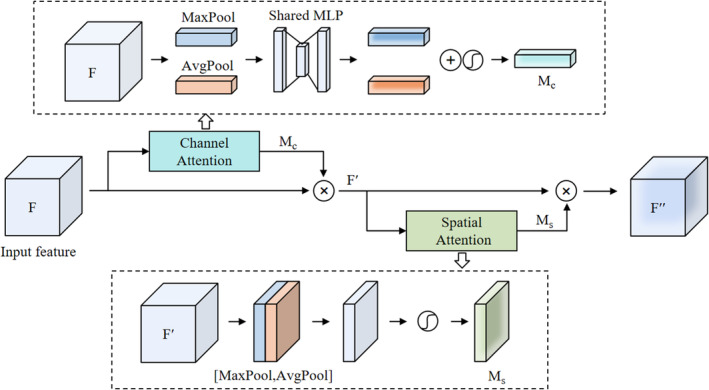
The workflow of CBAM architecture.

CBAM, as shown in Figure 3, consists of two sequential submodules: channel attention and spatial attention. Given the input feature $F\in R^{C\times H\times W}$, channel attention module applies max‐pooling and average‐pooling operations to generate two different spatial context descriptors $F_{\mathrm{avg}}^{c}$ and $F_{\max}^{c}$, followed by a shared MLP to generate a channel attention map $M_{c}\left(F\right)\in R^{C\times 1\times 1}$. This map is multiplied with the feature *F* to emphasise important channels. The operations in channel attention module can be formulated as follows:

(8) $$\begin{array}{c} M_{c}\left(F\right)=\sigma \left(\mathrm{MLP}\left(\text{AvgPool}\left(F\right)\right)+\mathrm{MLP}\left(\text{MaxPool}\left(F\right)\right)\right)\\ =\sigma \left(W_{1}\left(W_{0}\left(F_{\mathrm{avg}}^{c}\right)\right)+W_{1}\left(W_{0}\left(F_{\max}^{c}\right)\right)\right) \end{array},$$

(9) $$F^{\prime }=M_{c}\left(F\right)⊗F,$$

where *σ* denotes the sigmoid function, $W_{0}\in R^{C/r\times C}$ and $W_{1}\in R^{C\times C/r}$. It is important to know that MLP weights, that is, $W_{0}$ and $W_{1}$, are shared for both inputs, and ReLU activation function is followed by $W_{0}$. For spatial attention module, which applies max‐pooling and average‐pooling operations to input feature $F^{\prime }$, producing two features: ${F^{\prime }}_{\mathrm{avg}}^{s}$ and ${F^{\prime }}_{\max}^{s}$. These features are then processed by a convolution layer to generate a spatial attention map $M_{s}\left(F^{\prime }\right)\in R^{1\times H\times W}$, which is subsequently multiplied with channel‐refined feature $F^{\prime }$ to enhance important spatial features. In short, the spatial‐refined feature $F^{\prime \prime }$ is computed as:

(10) $$\begin{array}{c} M_{s}\left(F^{\prime }\right)=\sigma \left(f^{7\times 7}\left(\left[\text{AvgPool}\left(F^{\prime }\right);\text{MaxPool}\left(F^{\prime }\right)\right]\right)\right)\\ =\sigma \left(f^{7\times 7}\left(\left[{F^{\prime }}_{\mathrm{avg}}^{s};{F^{\prime }}_{\max}^{s}\right]\right)\right) \end{array},$$

(11) $$F^{\prime \prime }=M_{s}\left(F^{\prime }\right)⊗F^{\prime },$$

where *σ* denotes the sigmoid function and $f^{7\times 7}$ represents a convolution operation with filter size of 7 × 7.

### 2.5 MCBAM

**FIGURE 4:**
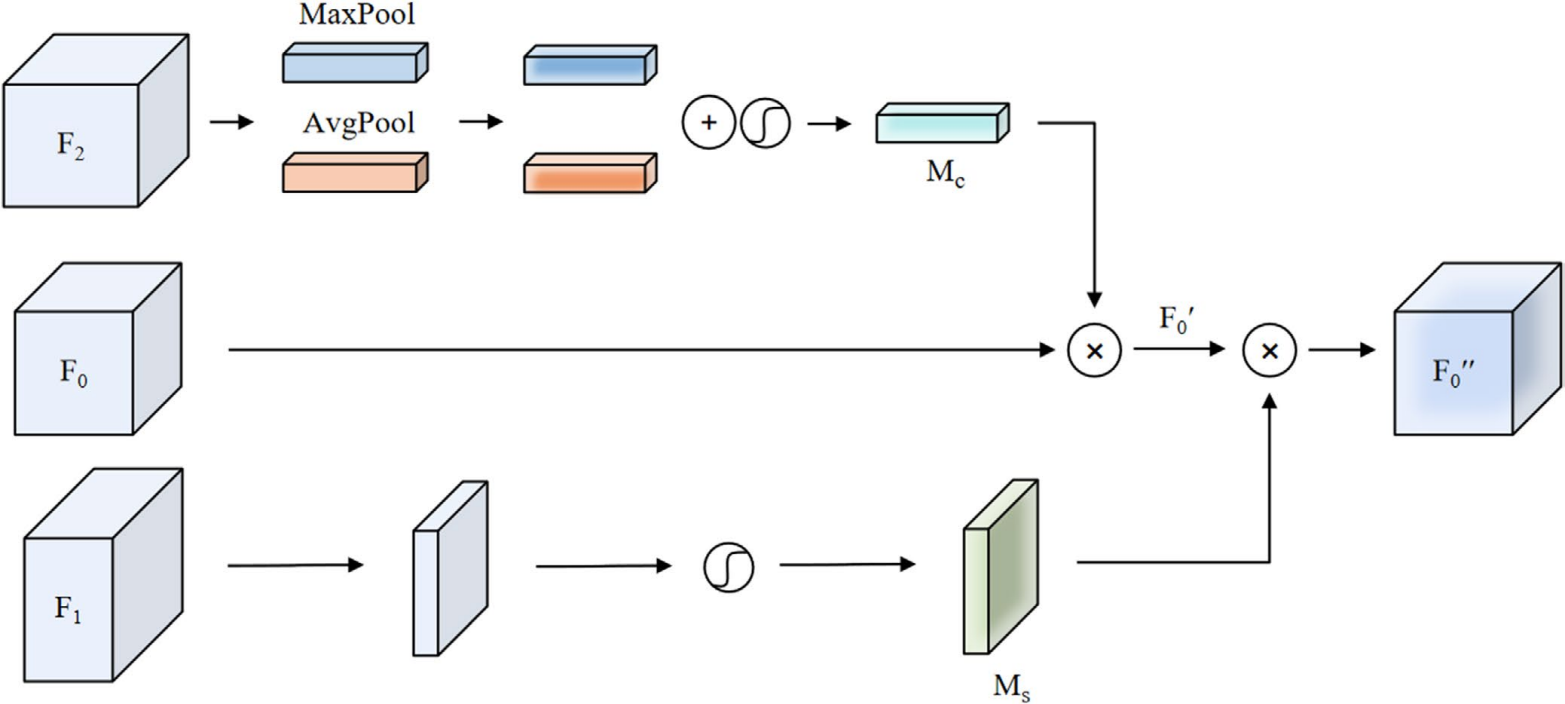
The architecture of MCBAM.

Building on the foundation of CBAM, MCBAM introduces the concept of channel and spatial attention modules to capture a richer set of features across different resolutions, as illustrated in Figure 4. This module processes features from multiple scales, including shallow features $F_{1}$ with dimension $C_{1}\times H_{1}\times W_{1}$, current layer features $F_{0}$ with dimension $C_{0}\times H_{0}\times W_{0}$ and deep features $F_{2}$ with dimension $C_{2}\times H_{2}\times W_{2}$.

$F_{2}$ are utilised for channel attention due to their rich semantic information and higher number of channels $C_{2}$, which can capture more complex inter‐channel relationships. MCBAM first applies max‐pooling and average‐pooling operations to$F_{2}$, resulting in two features of dimensions $C_{2}\times 1\times 1$. These pooled features are then fed into a convolution operation, transforming them into two new features of dimensions $C_{0}\times 1\times 1$. These two features are element‐wise added and passed through a sigmoid activation function to produce the channel attention map $M_{c}\left(F_{2}\right)\in R^{C_{0}\times 1\times 1}$. These weights are then multiplied element‐wise with $F_{0}$, resulting in a channel‐refined feature $F_{0}^{\prime }$.

For shallow features $F_{1}$, which predominantly capture low‐level visual information, there is a lack of rich semantic content and an increased presence of noise. To address this, we employ convolution and batch normalisation to re‐extract the shallow feature, generating a new feature with the dimension $1\times H_{0}\times W_{0}$. This process is followed by a sigmoid activation function to generate the spatial attention map $M_{s}\left(F_{1}\right)\in R^{1\times H_{0}\times W_{0}}$. It is then element‐wise multiplied with $F_{0}^{\prime }$, resulting in the final spatially enhanced feature $F_{0}^{\prime \prime }$.

### 2.6 Skip Connection

The skip connections are used to fuse the multi‐scale features from the encoder with the upsampled features in the decoder. In our architecture, skip connections are integrated with MCBAM to enhance feature representation further. Specifically, each MCBAM is designed to receive inputs from the current layer's encoding block. Features from the preceding encoding block are used as shallow features, while features from the succeeding encoding block are used as deep features. The output of MCBAM is a refined feature map that serves as the input for the skip connection. Notably, because there is no succeeding encoding block for the last encoding block, the output features from this block are processed through a CBAM before being fed into the decoder.

### 2.7 Decoder

Corresponding to the encoder, the symmetric decoder is built using MaxViT blocks. In contrast to MBConv layers used in the encoder for downsampling, decoder employs patch expanding layers to upsample the extracted deep features. The first three patch expanding layers reshape the feature maps into a higher resolution feature map, performing 2× upsampling while simultaneously reducing the channel dimension to half of its original value. Except for the final patch expanding layer, each is immediately followed by a skip connection that merges the upsampled features with the corresponding features from MCBAM. As the decoder processes the features through the first three patch expanding layers, the spatial dimensions are progressively restored from $\frac{H}{32}\times \frac{W}{32}\times 8C$ to $\frac{H}{4}\times \frac{W}{4}\times C$. Notably, the final patch expanding layer performs a 4× upsampling to restore the spatial resolution to match that of the original input image, producing an output with pixel‐level segmentation predictions.

## 3 Experimental Results

To validate the effectiveness of MSA‐MaxNet, we conduct a series of experiments using Synapse and ACDC datasets. This section provides detailed information on the dataset used, evaluation metrics and training strategies and presents results from both comparative and ablation experiments.

### 3.1 Datasets

Synapse comprises 3779 axial abdominal clinical CT images across 30 cases. Following the established protocols of the dataset, we divide these into 18 training and 12 testing samples. This dataset includes detailed annotations for eight abdominal organs, specifically the aorta, gallbladder, spleen, left kidney, right kidney, liver, pancreas and stomach.

ACDC dataset consists of MRI images from various patients, with labels for the left ventricle (LV), right ventricle (RV) and myocardium (Myo). This dataset is segmented into 70 training, 10 validation and 20 testing samples.

Kvasir‐SEG dataset comprises 1000 endoscopic polyp images and their corresponding ground truth segmentation masks.

### 3.2 Evaluation Metrics

On Synapse dataset, segmentation performance is evaluated using both average Dice similarity coefficient (DSC) and Hausdorff distance (HD95). In contrast, ACDC dataset is assessed solely with average DSC metric. For Kvasir‐SEG dataset, we comprehensively measure segmentation performance using DSC and Intersection over Union (IoU). DSC and IoU measure the overlap between the predicted segmentation and the ground truth. HD95 measures the 95th percentile of the maximum distance between the predicted segmentation boundary and the ground truth boundary, thus providing a measure of the largest segmentation error with reduced sensitivity to outliers. The formulas of metrics used in evaluation are as follows:

(12) $$\mathrm{DSC}=\frac{2|X\cap Y|}{|X|+|Y|},$$

(13) $$\mathrm{IoU}=\frac{|X\cap Y|}{|X\cup Y|},$$

(14)

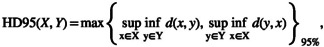

where *X* and *Y* are the sets of predicted and ground truth segmentation pixels respectively. The term ∣X∩Y∣ is the number of pixels common to both, while ∣X∣ and ∣Y∣ are the total number of pixels in the predicted and ground truth segmentations. The function $d\left(x,y\right)$ denotes Euclidean distance between points *x* and *y*, with *sup* and *inf* representing the supremum (maximum) and infimum (minimum) operators respectively. HD95 calculates Hausdorff distance by excluding the top 5% of the largest distance values, thus reducing the influence of outliers.

### 3.3 Training Strategies

Our model is trained based on Python 3.8 and PyTorch 2.0.0. For all training cases, data augmentations such as flips and rotations are used to increase data diversity. The training is conducted on an RTX 3090 GPU with 24 GB of memory. We employ the popular AdamW optimiser with a learning rate of 2e‐4 and a weight decay of 1e‐4, and a batch size of 8. All dataset splitting strategies, data processing methods and evaluation metrics strictly follow the standards established by previous works.

### 3.4 Comparative Experiments

In this section, we present a comparative analysis of MSA‐MaxNet against several leading models for multi‐organ CT segmentation, cardiac MRI segmentation and polyp pathology segmentation, demonstrating its effectiveness across different medical imaging modalities.

**FIGURE 5:**
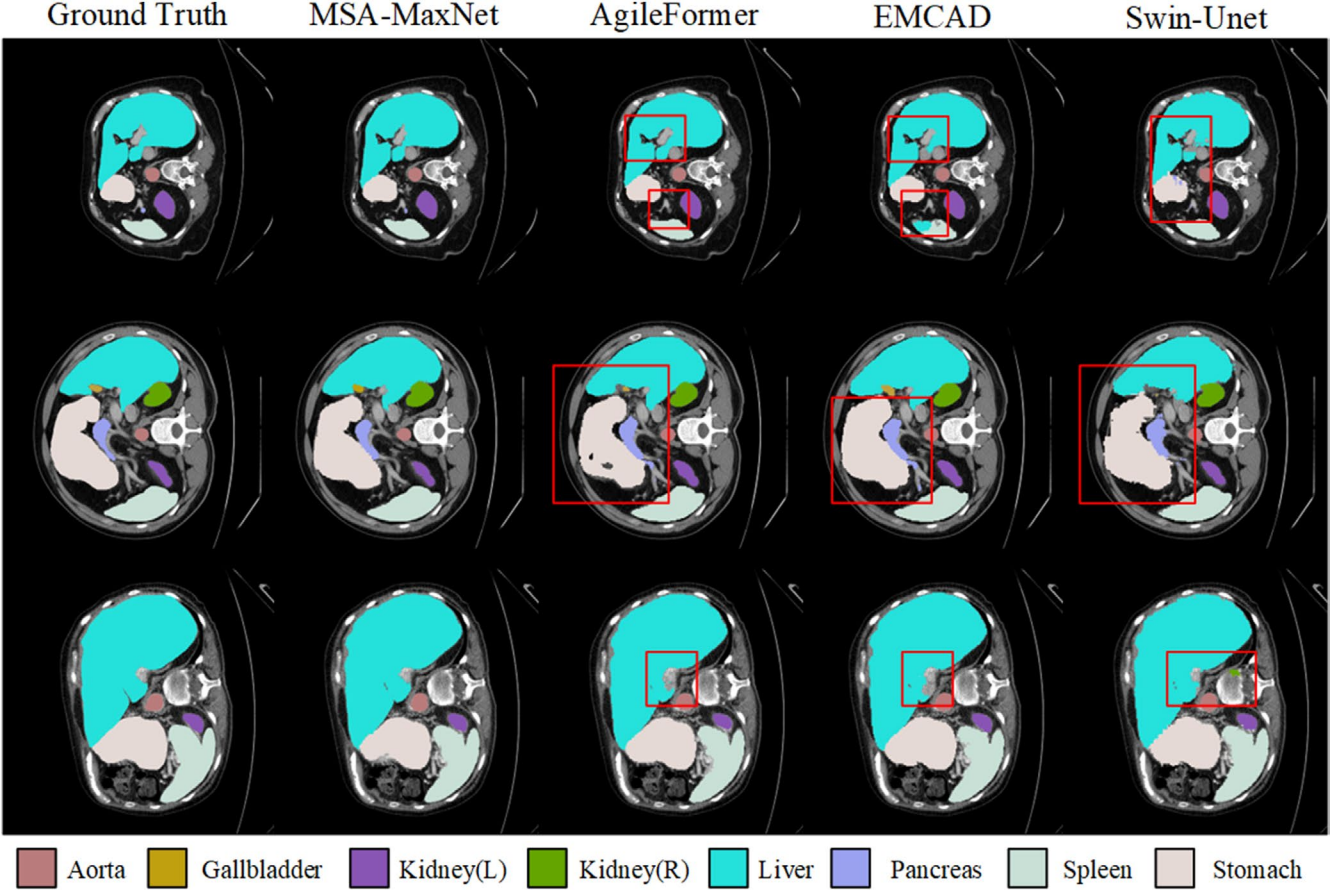
The segmentation results of different methods on Synapse. The division of the eight organs is represented by eight colours. Areas highlighted by the red box indicate where other models show clear deficiencies in segmentation compared to the ground truth.

The comparison of MSA‐MaxNet with other leading methods for multi‐organ segmentation on Synapse dataset is presented in Table 1. MSA‐MaxNet achieves an average DSC of 85.95%, surpassing previous SOTA models AgileFormer by 0.21% with three times fewer parameters. Notably, even when compared to EMCAD, which is renowned for its low parameter count, the small version of MSA‐MaxNet exceeds EMCAD by 0.84% in DSC while using approximately 20% fewer parameters. For weeny organs with complex boundaries, such as the aorta, gallbladder and pancreas, MSA‐MaxNet achieves notable improvements in DSC scores, demonstrating its enhanced adaptability to complex segmentation tasks. A qualitative comparison of different methods is illustrated in Figure 5 to corroborate its excellent performance. Additionally, compared to other models, MSA‐MaxNet demonstrates superior segmentation accuracy for larger organs such as the stomach, liver and spleen, offering more steady boundary definition and fewer hole errors.

**TABLE 1:** The comparison of other advanced networks with MSA‐MaxNet on Synapse.

Methods	Param (M)	DSC↑	HD95↓	Aorta	Gallbladder	Kidney (L)	Kidney (R)	Liver	Pancreas	Spleen	Stomach
MSA‐MaxNet	40.28	**85.95**	14.59	**90.80**	**78.22**	86.19	84.45	95.72	**72.66**	**93.21**	**86.31**
MSA‐MaxNet (Small)	21.72	84.47	**12.41**	89.80	74.13	85.67	82.28	**95.76**	70.00	91.99	86.10
MSA‐MaxNet (Tiny)	9.83	82.66	20.17	89.44	73.70	80.40	79.95	95.34	67.50	92.97	81.97
AgileFormer [33]	113.0	85.74	7.81	89.11	77.89	**88.83**	**85.00**	95.64	71.62	92.20	85.63
MERIT [39]	147.9	84.90	13.22	87.71	74.40	87.79	84.85	95.26	71.81	92.01	85.38
EMCAD [36]	26.76	83.63	15.68	88.14	68.87	88.08	84.10	95.26	68.51	92.17	83.92
MISSFormer [40]	42.46	81.96	18.20	86.99	68.65	85.21	82.00	94.41	65.67	91.26	80.81
Swin‐UNet [31]	27.17	79.13	21.55	85.47	66.53	83.28	79.61	94.29	56.58	90.66	76.60
UCTransnet [35]	—	78.74	28.87	88.49	65.49	83.54	76.39	93.91	57.07	90.34	74.72
TransUnet [30]	96.07	77.48	31.69	87.23	63.13	81.87	77.02	94.08	55.86	85.08	75.62
Att‐UNet [25]	—	77.77	36.02	89.55	68.88	77.98	71.11	93.57	58.04	87.30	75.75
U‐Net [23]	20.01	76.85	39.70	89.07	69.72	77.77	68.60	93.43	53.98	86.67	75.58

*Note:* The table showcases the parameter count for each model (in millions, M), average DSC, average HD95 and DSC segmentation performance for each organ. Bolded text indicates the highest score achieved for organ segmentation.

Experimental results on ACDC dataset, shown in Table 2, demonstrate that MSA‐MaxNet consistently outperforms the currently established advanced methods in cardiac MRI segmentation tasks. Overall, MSA‐MaxNet achieves a DSC of 92.36%, surpassing the second‐best model EMCAD by 0.24%. Additionally, MSA‐MaxNet achieves the highest segmentation performance for both the myocardium and the left ventricle, with its performance on the right ventricle being only 0.02% lower than that of the MERIT model.

**TABLE 2:** The segmentation accuracy of leading methods on ACDC dataset.

Methods	DSC	RV	Myo	LV
MSA‐MaxNet	**92.36**	90.85	**90.08**	**96.15**
MSA‐MaxNet (Small)	91.74	89.91	89.43	95.87
MSA‐MaxNet (Tiny)	91.26	89.27	88.86	95.66
MERIT [39]	92.32	**90.87**	90.00	96.08
EMCAD [36]	92.12	90.65	89.68	96.02
PVT‐GCASCADE [41]	91.95	90.31	89.63	95.91
Swin‐UNet [31]	90.00	88.55	85.62	95.83
TransUnet [30]	89.71	88.86	84.53	95.73

*Note:* The average DSC and the DSC scores for RV (right ventricle), Myo (myocardium) and LV (left ventricle) are reported. The bold values represent the best performace for evaluation metrics.

Table 3 presents a comparison of the segmentation performance of MSA‐MaxNet on the Kvasir‐SEG dataset. MSA‐MaxNet achieves a DSC of 95.26%, surpassing SOTA models DUCK‐Net 0.24% in DSC and 0.71% in IoU metrics, demonstrating high overlap, low error rate and strong robustness. Furthermore, the tiny version of MSA‐MaxNet outstrips all other models including DUCK‐Net.

**TABLE 3:** The segmentation accuracy of SOTA methods on Kvasir‐SEG dataset.

Methods	DSC↑	IoU↑
MSA‐MaxNet	**95.26**	91.24
MSA‐MaxNet (Small)	95.22	**91.25**
MSA‐MaxNet (Tiny)	95.09	91.00
DUCK‐Net [42]	95.02	90.51
EffiSegNet‐B5 [43]	94.88	90.65
FCB Former [44]	94.45	—
EMCAD [36]	92.80	—

*Note:* The average DSC and IoU are reported. The bold values represent the best performace for evaluation metrics.

### 3.5 Ablation Experiments

To systematically assess the individual contributions of each key component within MSA‐MaxNet, we conduct a series of experiments on Synapse dataset, focusing on downsampling methods, attention mechanisms and skip connection variations. The results of the ablation experiment are shown in Table 4. In the ablation experiment, we create MSA‐MaxNet‐2 by replacing MBConv modules with standard convolutional layers, leading to a 1.25% decrease in DSC score. This result underscores the essential role of MBConv in enhancing feature extraction during the downsampling process. To evaluate the impact of different attention mechanisms, we develop MSA‐SwinNet by substituting MaxViT blocks with Swin Transformer blocks, which produced a notable 4.03% drop in DSC, highlighting the importance of block and grid attention in the encoder–decoder structure. We also examine the effect of our innovative skip connection design by comparing the full MSA‐MaxNet with a variant MaxNet that excludes any modification on skip connections. MaxNet sees a 1.69% decrease in DSC, justifying the critical function of skip connection in facilitating multi‐scale information flow and robust feature representation. Finally, we assess the effectiveness of MCBAM by replacing it with CBAM to create CA‐MaxNet. However, the DSC score of CA‐MaxNet is 0.72% lower than MSA‐MaxNet, indicating the superior feature refinement capabilities of MCBAM. Collectively, the consequence of ablation studies affirms the positive impact of MSA‐MaxNet's components in optimising segmentation performance.

**TABLE 4:** Comparative analysis of MSA‐MaxNet and its variants in ablation experiments conducted on Synapse dataset.

Methods	Downsampling		Encoding (Decoding)		Skip connections		DSC↑	HD95↓
	MBConv	Conv	MaxVit	SwinT	MCBAM	CBAM		
MSA‐MaxNet	√		√		√		**85.95**	**14.59**
MSA‐MaxNet‐2		√	√		√		84.70	17.36
MSA‐SwinNet	√			√	√		81.92	21.53
CA‐MaxNet	√		√			√	85.23	17.96
MaxNet	√		√				84.26	17.88

*Note:* √ means the component included in the ablation models. The bold values represent the best performace for evaluation metrics.

## 4 Discussion and Conclusion

### 4.1 Applications in Biology and Medicine

Medical image segmentation has become a fundamental tool in advancing molecular and cellular biology, as well as in clinical applications such as pathology and personalised medicine. In particular, deep learning‐based segmentation methods, like MSA‐MaxNet, hold great promise for revolutionising the way we analyse and interpret complex biological and medical images.

One compelling application lies in the study of cellular processes at a molecular level. For example, accurate segmentation of cellular components in microscopy images could enable high‐throughput screening of disease biomarkers, facilitating early‐stage disease detection and providing insights into cellular behaviour under different conditions. This is particularly important in cancer research, where early detection of malignant tissues can significantly improve treatment outcomes. Similarly, in molecular biology, segmentation techniques can aid in identifying and quantifying specific structures like proteins, organelles and nucleic acids within cells, enabling a better understanding of cellular processes, such as gene expression and protein synthesis.

In pathology, segmentation plays a crucial role in the diagnosis and prognosis of diseases. Precise segmentation of tissues in histological images allows pathologists to identify abnormal growth patterns, tissue heterogeneity and microenvironment changes, which are essential for accurate diagnosis, staging and treatment planning. In particular, the segmentation of cancerous regions in tissue biopsies can support pathologists in identifying early‐stage tumours, which might otherwise be difficult to detect due to their small size or complex morphology. Additionally, segmentation of different tissue types, such as muscle, fat and blood vessels, in medical imaging helps in the quantitative assessment of tissue composition, which can further aid in diagnosing diseases like heart disease, liver fibrosis or neurodegenerative conditions.

The integration of advanced segmentation models like MSA‐MaxNet into pathology workflows could further enhance the standardisation of diagnostic processes, enabling automated, reproducible and precise analyses. By reducing the burden on pathologists and providing consistent, accurate results, these models can improve diagnostic accuracy and help in the identification of subtle pathological patterns, which may be crucial for prognosis and personalised treatment strategies.

### 4.2 Summary and Future Outlook

The continuous advancements in medical imaging and machine learning have significantly enhanced the capabilities of medical image segmentation, particularly in critical applications such as the diagnosis of major diseases, and structural tissue analysis. Accurate and efficient segmentation is crucial for improving diagnostic precision and treatment planning. In this work, we present MSA‐MaxNet, a novel approach that enhances segmentation performance by combining multi‐axis self‐attention mechanisms with an encoder–decoder structure and an innovative skip connection module. Experimental results demonstrate that MSA‐MaxNet achieves DSC scores of 85.95%, 92.36% and 95.26% on Synapse, ACDC and Kvasir‐SEG datasets, respectively, showcasing its leading position among current segmentation models. Especially in the challenges of multi‐organ and polyp segmentation, MSA‐MaxNet sets a SOTA benchmark. This not only validates its outstanding potential in tackling complex medical image segmentation tasks but also highlights its significant application value in pathology, such as the precise identification of early cancerous regions, structural tissue analysis quantitative analysis of pathological features, and efficient assistance in pathological diagnostic workflows.

The superiority of MSA‐MaxNet can be attributed to its unique architecture that combines the strengths of UNet framework with the advanced capabilities of Max‐SA. This integration allows our model to effectively capture local features by block attention while interacting with semantic information globally and over long distances through grid attention. Simultaneously, our custom‐designed MCBAM utilises channel and spatial attention mechanisms to refine feature fusion and representation by aggregating multi‐scale features from hierarchical convolution. These innovations enable MSA‐MaxNet to effectively address segmentation challenges such as false positive segmentation, poor segmentation accuracy, blurred and incomplete edge segmentation for large organs and noise and poor shape prediction for smaller structures.

Despite the promising results, there are several limitations and areas for future improvement. MSA‐MaxNet has relatively high computational complexity due to its ViT‐based architecture. Since CNNs were introduced earlier and have undergone extensive optimisation for modern hardware, MSA‐MaxNet exhibits slightly slower inference speeds compared to purely CNN‐based networks. To address this, we may explore new lightweight modules, parameter‐sharing mechanisms or sparse connections to reduce computational load while maintaining, or even enhancing, segmentation accuracy. Additionally, the lack of validation on clinically representative datasets highlights the need for further testing to ensure robustness and applicability in real‐world scenarios. Addressing these limitations is especially critical in pathology, where imaging tasks often present unique challenges, such as high‐resolution requirements, diverse staining protocols and the need to identify intricate tissue structures. With its ability to balance local and global feature extraction, MSA‐MaxNet has the potential to tackle these complexities. Future research could explore its deeper applications in advancing cancer detection, assessing pathological biomarkers and offering detailed insights into tissue‐level morphology. Incorporating MSA‐MaxNet into digital pathology workflows may help standardise assessments, reduce workload and uncover subtle pathological patterns. Further efforts should focus on validating its performance on high‐resolution pathological datasets and integrating it with advanced imaging modalities, such as multiplex immunofluorescence and hyperspectral imaging, to expand its utility in precision medicine.

## Author Contributions

**Wei Wu:** conceptualization (equal), data curation (equal), formal analysis (equal), investigation (equal), writing – original draft (equal). **Junfeng Huang:** data curation (equal), formal analysis (equal), investigation (equal), writing – original draft (equal). **Mingxuan Zhang:** software (equal), validation (equal), visualization (equal). **Yichen Li:** software (equal), validation (equal), visualization (equal). **Qijia Yu:** software (equal), validation (equal), visualization (equal). **Qi Zhao:** conceptualization (lead), funding acquisition (lead), methodology (lead), project administration (lead), resources (lead), supervision (lead), writing – original draft (lead), writing – review and editing (lead).

## Conflicts of Interest

The authors declare no conflicts of interest.

## Data Availability

The source code is available online at https://github.com/zhaoqi106/MSA‐MaxNet.
